# OA14 The role of anifrolumab in treating life threatening systemic lupus erythematosus with severe mucocutaneous involvement

**DOI:** 10.1093/rap/rkad070.014

**Published:** 2023-09-27

**Authors:** Christopher Baldwin, Sasha Ali, Deepak Nagra, Jennifer Hannah, Patrick Gordon, James Galloway, Chris Wincup

**Affiliations:** King's College Hospital, London, United Kingdom; King's College Hospital, London, United Kingdom; King's College London, London, United Kingdom; King's College Hospital, London, United Kingdom; King's College London, London, United Kingdom; King's College Hospital, London, United Kingdom; King's College London, London, United Kingdom; King's College Hospital, London, United Kingdom; King's College London, London, United Kingdom; King's College Hospital, London, United Kingdom; King's College London, London, United Kingdom; King's College Hospital, London, United Kingdom

## Abstract

**Introduction:**

Systemic lupus erythematosus (SLE) refractory to conventional DMARDs and anti-malarials has limited treatment options including rituximab, cyclophosphamide and belimumab. Although not currently licensed for use in the UK, anifrolumab (a monoclonal antibody targeting the type 1 interferon receptor) has successfully reached primary endpoints in clinical trials of moderate-to-severe disease. Whilst trials have shown benefit in skin disease, the role of the drug in severe cutaneous manifestations of SLE is currently unclear. Herein, we present the case of a patient with severe, life-threatening cutaneous SLE refractory to mycophenolate, rituximab and cyclophosphamide successfully treated with anifrolumab.

**Case description:**

A 47-year-old woman, diagnosed with SLE in 2015, presented with a significant disease flare. Previous manifestations included cutaneous disease, alopecia, inflammatory arthritis, autoimmune hepatitis, and pulmonary/mesenteric vasculitis. She had received recent aggressive therapy with mycophenolate, cyclophosphamide and rituximab (achieving peripheral B cell depletion).

On this occasion, she displayed severe cutaneous features including a diffuse erythematosus rash involving the arms and trunk, and livedo reticularis on both legs. She also had extensive oral ulceration, marked alopecia, inflammatory synovitis and breathlessness at rest. Blood tests demonstrated raised anti-dsDNA antibodies (>400 IU/ml) with low complement C3 (0.37 g/L) and C4 (0.04 g/L). Imaging confirmed the presence of bilateral pleural effusions and lupus enteritis. SLEDAI-2K on admission was 29.

Initial management comprised of intravenous methylprednisolone followed by IV cyclophosphamide (Euro-Lupus protocol). Due to recurrent infections (warranting multiple admissions to the ITU for respiratory/ionotropic support for severe sepsis), cyclophosphamide was suspended after 2 doses. Balancing active SLE and severe infection posed significant challenges and high doses of steroids were used. Significantly raised serum CXCL10 (1788pg/ml) suggested the disease was interferon driven, so anifrolumab was considered.

Due to recurrent infections she was ineligible for clinical trials of anifrolumab. However, access was granted on compassionate grounds and she started at a dose of 300mg IV every 4 weeks. This resulted in a significant improvement in disease activity with anti-dsDNA titres improved to 230 IU/ml and C3 complement normalised. Her skin completely healed and alopecia entirely resolved. After a 309 day admission, she was discharged home with a SLEDAI-2K of 2 and having weaned prednisolone to 7.5 mg a day, thus fulfilling Lupus Low Disease Activity State (LLDAS) on discharge.

Of note, she was vaccinated against herpes zoster immediately prior to treatment but still did develop a breakthrough shingles infection.

**Discussion:**

Despite mycophenolate, rituximab and cyclophosphamide, this patient had persisting significantly active disease, thus highlighting the challenges of managing refractory cutaneous/multisystem SLE. Although NICE guidelines recommend the use of belimumab in high disease activity (SLEDAI-2K ≥10), concerns were raised due to the slow onset of clinical benefit in clinical trials and lack of evidence in severe cutaneous disease. We noted CXCL10 (a chemokine induced by interferon and therefore believed to be a potential surrogate marker for interferon activity) was significantly raised, suggesting interferon to be a key driver of disease in this instance. Anifrolumab regulates clonal population sizes of both B and T cells via inhibiting type 1 interferon receptor. Additionally, she had previously shown poor response to B cell directed therapy with rituximab. Belimumab is a monoclonal antibody directed against BAFF that mediates the survival and differentiation of B cells and their differentiation. Therefore, mechanism of action of anifrolumab was felt to be preferable to belimumab.

In the TULIP 2 study, anifrolumab demonstrated a 47.8% improvement in disease activity by BICLA (BILAG-Based Composite Lupus Assessment) response vs 31.5% in placebo. TULIP 2 demonstrated a greater than 50% improvement in CLASI (Cutaneous lupus disease area and severity index) in 49% of patients receiving anifrolumab compared to 25% in placebo. Furthermore, TULIP 2 also demonstrated a significant, sustained reduction in glucocorticoid use in anifrolumab relative to placebo (51.5% vs 30.2%).

The clinical trials noted an increased risk of herpes zoster infection with anifrolumab compared to placebo, and routine vaccination against herpes zoster should be considered in patients starting anifrolumab. In our case this did not prevent breakthrough infection.

Although not currently available via NICE in the UK, we feel this case demonstrates the potential of anifrolumab in the treatment of severe multi-system disease, including severe cutaneous manifestations of SLE, in the future.

**Key learning points:**

SLE can be a challenging to treat, and in severe, life-threatening cases there are limited treatment options available, particularly those targeting specific immune pathways. It has been shown that molecular and immunological phenotyping stratifies SLE into several major groups that may help explain this variable treatment response. We have highlighted a patient with poor clinical response to mycophenolate, cyclophosphamide and rituximab with life threatening lupus and extremely active mucocutaneous disease, deriving significant benefit from anifrolumab.

Initial trials involving anifrolumab showed promising results in terms of overall disease activity, reducing severity of skin activity and decreasing glucocorticoid burden. In this case, the patient continued to have profoundly active disease, despite using several conventional available therapies. Following a protracted inpatient stay (309 days), with more than six admissions to ITU, anifrolumab provided an additional avenue of treatment and subsequently induced almost complete disease remission and significantly decreased steroid burden. Although this patient’s disease was severely active (and given the complication of recurrent infections would therefore make it unlikely she would have been considered as suitable for clinical trials of anifrolumab), the response appears consistent with the outcomes of the TULIP 2 study.

Clinical trials also noted an increased risk of herpes zoster infection with anifrolumab and it may be suggested that routine vaccination should be considered in patients starting this treatment. In this case, the patient was vaccinated against herpes zoster immediately prior to treatment but still developed a mild breakthrough infection across a single dermatome.

This case also demonstrates the potential use of CXCL10 as a biomarker for interferon activity, and its subsequent role in the selection of a biologic (anifrolumab) targeting this specific pathway. Future studies are required to evaluate as to whether this is a useful marker for stratifying therapy or predicting response to interferon targeting therapies.

